# Mapping the Landscape of Digital Health Intervention Strategies: 25-Year Synthesis

**DOI:** 10.2196/59027

**Published:** 2025-01-13

**Authors:** Shiyu Liu, Jingru Ma, Meichen Sun, Chao Zhang, Yujing Gao, Jinghong Xu

**Affiliations:** 1 School of Public Health Xi’an Jiaotong University Xi'an China; 2 School of Pharmacy Shanghai University of Traditional Chinese Medicine Shanghai China; 3 School of Journalism and Cultural Communication Zhongnan University of Economics and Law Wuhan China; 4 School of Journalism and Communication Beijing Normal University Beijing China; 5 The International College Krirk University Bangkok Thailand

**Keywords:** digital health interventions, intervention strategies, behavior change, mHealth, eHealth, randomized controlled trial

## Abstract

**Background:**

Digital health interventions have emerged as promising tools to promote health behavior change and improve health outcomes. However, a comprehensive synthesis of strategies contributing to these interventions is lacking.

**Objective:**

This study aims to (1) identify and categorize the strategies used in digital health interventions over the past 25 years; (2) explore the differences and changes in these strategies across time periods, countries, populations, delivery methods, and senders; and (3) serve as a valuable reference for future researchers and practitioners to improve the effectiveness of digital health interventions.

**Methods:**

This study followed a systematic review approach, complemented by close reading and text coding. A comprehensive search for published English academic papers from PubMed, Web of Science, and Scopus was conducted. The search employed a combination of digital health and intervention-related terms, along with database-specific subject headings and filters. The time span covered 25 years, from January 1, 1999, to March 10, 2024. Sample papers were selected based on study design, intervention details, and strategies. The strategies were identified and categorized based on the principles of Behavior Change Techniques and Behavior Strategies.

**Results:**

A total of 885 papers involving 954,847 participants met the eligibility criteria. We identified 173 unique strategies used in digital health interventions, categorized into 19 themes. The 3 most frequently used strategies in the sample papers were “guide” (n=492, 55.6%), “monitor” (n=490, 55.4%), and “communication” (n=392, 44.3%). The number of strategies employed in each paper ranged from 1 to 32. Most interventions targeted clients (n=844, 95.4%) and were carried out in hospitals (n=268, 30.3%). High-income countries demonstrated a substantially higher number and diversity of identified strategies than low- and middle-income countries, and the number of studies targeting the public (n=647, 73.1%) far exceeded those focusing on vulnerable groups (n=238, 26.9%).

**Conclusions:**

Digital health interventions and strategies have undergone considerable development over the past 25 years. They have evolved from simple approaches to sophisticated, personalized techniques and are trending toward multifaceted interventions, leveraging advanced technologies for real-time monitoring and feedback. Future studies should focus on rigorous evaluations, long-term effectiveness, and tailored approaches for diverse populations, and more attention should be given to vulnerable groups.

## Introduction

### The Rise of Digital Health Interventions

The rapid advancement of digital technologies has significantly influenced health practices. As of 2020, digital health has more than 90 definitions, encompassing eHealth, mHealth, self-tracking, wearable devices, and artificial intelligence applications [1]. Digital health interventions, defined as interventions delivered via digital health technologies, have emerged as promising tools for promoting healthy behaviors and improving health outcomes [2]. The effectiveness of digital health interventions has been validated across diverse domains, populations, and countries. Specifically, digital health interventions have demonstrated success in multiple health areas, such as noncommunicable disease management [3], mental health support [4], and smoking cessation programs [5]. In addition, these interventions have proven effective in serving not only the general population but also vulnerable groups, such as resource-poor communities and ethnic minorities [6]. Notably, their application extends across geographical and economic boundaries, showing positive results in both high-income nations, such as the United States [7] and Canada [8], and resource-limited regions, including Pakistan [9] and West Africa [10].

Driven by technological innovations, increasing smartphone penetration, and the growing acceptance of digital health technologies, health care delivery is experiencing a significant transformation from traditional processes to digital interventions [11]. The COVID-19 pandemic has further accelerated this transformation [12]. As health care systems globally confront challenges, including aging populations, rising chronic disease burdens, and disparities in health care access, the adoption and integration of digital health interventions have become increasingly crucial [13]. Moreover, recent advances in artificial intelligence have further enhanced the importance and impact of digital health interventions.

### The Critical Role of Strategies in Digital Health Interventions

Strategies are indispensable for generating the desired effects of digital health interventions. Selecting and implementing appropriate strategies are crucial for ensuring their success [14]. Well-designed strategies have the potential to enhance user engagement, promote behavior change, and contribute to improved health outcomes [15,16], although their effectiveness may vary across different populations and contexts. Through the application of well-defined, theory-based strategies, digital health interventions can provide enhanced support for individuals in achieving their health goals, ultimately leading to more sustainable health care approaches [17].

Strategic approaches play a crucial role throughout the intervention process, from initial design to implementation. For example, message strategies, which form the core of the intervention design phase and are based on principles such as reciprocity, reciprocity-by-proxy, and curiosity, have proven effective in engaging racial and ethnic minority groups in digital oral self-care interventions [18]. During the implementation process, strategies can help overcome barriers to achieving intervention goals through four key approaches: (1) increasing coherence (ensuring the intervention is meaningful to users); (2) enhancing cognitive participation (securing user engagement); (3) promoting collective action (supporting the implementation process); and (4) enabling reflexive monitoring (facilitating an assessment of the intervention's effects) [19].

### Current Findings on Digital Health Intervention Strategies

Currently, several studies, including systematic reviews and meta-analyses, have explored the impact of various strategies on health behaviors and outcomes in digital health interventions. Saleem et al [20] found that engagement strategies including feedback, guidance, social support, content gamification, reminders, flexibility, and ease of use can promote mental health outcomes. Behavior change strategies including follow-up prompts, self-monitoring, emotional control training, and provision of information about others’ approval were widely used to contribute to health behavior [21].

A recent meta-analysis for weight management interventions found that strategies like problem-solving, goal setting, reviewing goals, feedback on behavior, self-monitoring of behavior, behavioral substitution, and credible sources were successful in addressing decreased energy [22]. The strategies of sustained motivation, self-regulation, psychological and physical resources, habit formation, and environmental and social influences were valued by weight loss maintenance intervention [23,24]. Four strategies, including improving social skills, enhancing social support, increasing social contact, and addressing cognition, have been identified to reduce loneliness [25]. Eleven intervention strategies, primarily involving medication and diet control, including physical activity, lipid-affecting drugs, and diet plus exercise, have been identified to prevent type 2 diabetes mellitus in China [26].

In addition, substantial strategies have been found to be effective in improving recruitment, reducing loss to follow-up, and enhancing retention [27-30]. A total of 72 strategies were tested to enhance participant recruitment in randomized trials, with 7 of these strategies being investigated in multiple studies [31]. Six broad types of strategies, including incentives, communication strategies, new questionnaire formats, participant case management, behavioral interventions, and methodological interventions, were summarized as effective approaches to improve retention [32]. Robinson et al [33] abstracted 368 strategies from 21 studies and identified 12 themes aimed at retaining participants. They found that contact and scheduling were the most common strategies to limit participant attrition in health care research.

### Study Objectives

The previous research on digital health intervention strategies [23-34], despite its comprehensiveness, has significant limitations, notably its narrow focus on specific health issues or populations and its inadequate exploration of behavior change. Moreover, numerous nuanced strategies remain unidentified and analyzed, requiring further investigation. Accordingly, it is crucial to identify, categorize, and aggregate strategies used in digital health interventions. As the field of digital health continues to evolve, a deeper understanding of intervention strategies will be vital for advancing both research and practice, offering valuable insights into their effectiveness and guiding the design of future interventions. By identifying and categorizing these strategies, researchers and practitioners can gain a clearer understanding of effective approaches and techniques in digital health interventions, evaluating the strengths and limitations of different strategies and their relevance to specific health domains or populations. Furthermore, a comprehensive understanding of digital health intervention strategies can support the development of evidence-based guidelines and best practices, fostering the standardization and quality of digital health interventions.

Therefore, this study aimed to (1) identify and categorize the strategies employed in digital health interventions over the past quarter century; (2) explore the differences and changes in these strategies across time periods, countries, populations, deliveries, and senders; and (3) develop a catalog of strategies to serve as a reference for future researchers and practitioners to improve the effectiveness of digital health interventions.

## Methods

### Overview

This article followed a systematic review approach, complemented by close reading and text coding. The systematic review was used to access and screen relevant literature, while textual close reading was employed to identify and categorize strategies. The identification and categorization of strategies were guided by the principles and catalogs of Behavior Change Techniques (BCTs) [35] and Behavior Strategies (BSs) [22]. This dual approach ensures a comprehensive and rigorous analysis of the strategies used in digital health interventions.

### Search Methods

Our research steps were guided by PRISMA-ScR (Preferred Reporting Items for Systematic Reviews and Meta-Analyses extension for Scoping Reviews) guidelines (Multimedia Appendix 1) [36]. We conducted a comprehensive search for published English papers from PubMed, Web of Science, and Scopus. These databases are globally esteemed for their broad interdisciplinary coverage, sophisticated search tools, and current updates, which ensure systematic reviews are comprehensive, accurate, and authoritative [37]. Based on these characteristics, we selected these 3 databases for the following reasons: (1) broad coverage: these databases offer extensive coverage of medical and health-related research, enabling us to capture a wide range of relevant studies; (2) interdisciplinary nature: the selected databases cover related fields such as psychology, public health, and information technology; (3) quality and reliability: these databases are recognized for their rigorous inclusion criteria and high-quality indexing.

This search employed a combination of digital health and intervention-related terms, along with database-specific subject headings and filters, to ensure thoroughness and focus. The detailed search strategy for each database can be found in Multimedia Appendix 2. The time span considered was the past 25 years, from January 1, 1999, to March 10, 2024.

### Selection of Sample Papers

We conducted 2 rounds of screening and selection. The first round focused on identifying digital health interventions, while the second round aimed to pinpoint specific strategies within those interventions.

For the first round of screening, pairs of reviewers (authors JM, MS, YG, NZ, ML, and HD), all graduate students majoring in public health or health communication, independently reviewed the titles, abstracts, and keywords of the sample articles to preliminarily identify studies that met our eligibility criteria. The specific screening criteria and steps were as follows: (1) the means of the intervention should be digital, primarily including wearable devices, telemedicine, electronic health records, electronic medical records, mobile phone applications, web pages, blogs, emails, text messages, social media, and similar technologies; (2) the intervention must be health-related, encompassing health behavior improvement, disease treatment, and health education, and so on. (3) the studies must be randomized controlled trials (RCTs), including individual and cluster RCTs, as they provide the highest level of evidence for evaluating interventions. We excluded randomized feasibility and pilot trials because they are inherently preliminary studies conducted to inform future RCTs [38].

**Figure 1:**
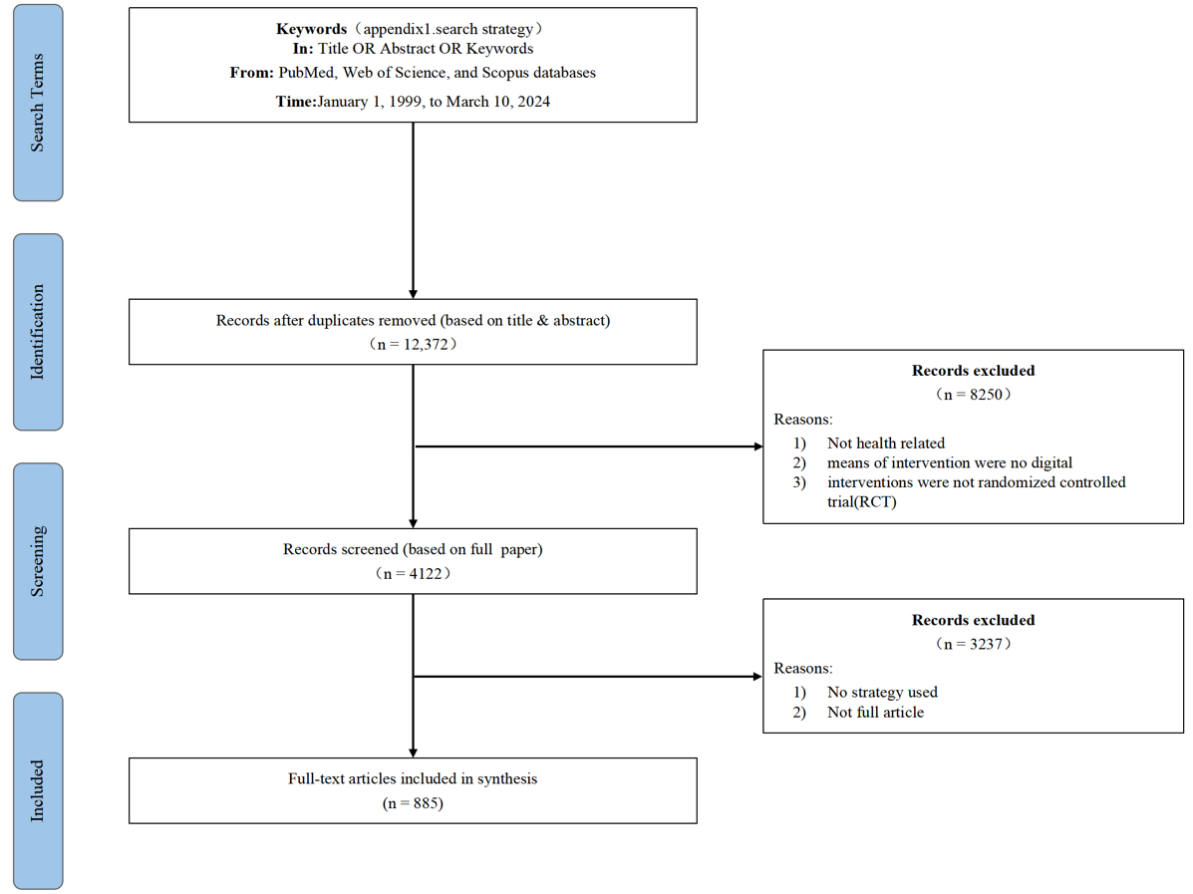
Flow diagram of literature search and screening.

In the second round of screening, we concentrated on identifying specific strategies within the selected digital health interventions. For this study, a strategy was defined as a specific approach or technique employed within the intervention to promote health behavior change or improve health outcomes. The second round of screening involved a full-text examination of the included sample papers. The reviewers (JM, MS, YG, NZ, ML, and HD) assessed the descriptions of the interventions and the strategies employed. The flow of literature searches and screening is shown in Figure 1.

To guide the strategy extraction, we provided a framework and definitions of potential strategies (Multimedia Appendix 3) organized into 18 clusters and 64 themes based on BCTs [35] and BSs [22]. Recognizing the possibility of strategies extending beyond these predefined themes, we incorporated an “other” theme to capture a broader range of strategies. This adaptable framework aimed to maximize the thoroughness and accuracy of the data collection process, ensuring that novel or unique strategies were not overlooked.

After identifying all strategies, we conducted a comprehensive review to pinpoint overarching themes and classify each strategy accordingly. Initially, 3 authors (SL, YG, and CZ) examined the strategies and proposed themes independently. Subsequently, the first author (SL) reviewed the independent results, reconciled discrepancies, and developed a list of common themes. Next, a team meeting discussed the proposed themes and strategy categorization, leading to a final list of themes and assigning each strategy to 1 category. Finally, the senior authors reviewed and adjudicated the assigned themes, with any disagreements resolved through team discussions.

We assessed interrater reliability across 2 rounds of the coding process. In the first round, 6 independent students screened articles based on titles, abstracts, and keywords. Due to the large volume of articles, a 1% sample was used for training purposes. The interrater reliability was measured using Fleiss kappa, yielding a coefficient of 0.82. In the second round, a coding guideline was developed, and 6 students underwent thorough training before proceeding with coding. Initially, 10% of the articles were used for calibration, resulting in a Krippendorff alpha of 0.85 for intercoder reliability. Inconsistencies were addressed through meetings, with a senior researcher arbitrating persistent disagreements. Any issues encountered during the coding process were promptly discussed in regular meetings and our WeChat group.

We recorded the number of articles identified, screened, deemed eligible, and ultimately included in the study, along with coding, using Microsoft Excel (IBM Corp). This systematic approach guaranteed a transparent process for capturing papers that met our inclusion criteria.

### Data Extraction and Coding

In this study, we coded several key aspects from the included sample papers. General information such as the journal of publication, authors, year of publication, the geographical context of the intervention, demographic characteristics of participants, and the theoretical framework or models underpinning the intervention (if reported) were documented.

We also examined the characteristics of the digital health interventions, including the health concerns they addressed, delivery modalities (eg, short text messages, smartphone apps, and online platforms), intervention duration, sender type (automated or human-assisted), and their classification according to the World Health Organization’s (WHO) 2018 framework (patients, health care professionals, health system administrators, and data services) [39]. Additionally, strategies were identified and labeled based on a structured framework and descriptive criteria established for this study (Multimedia Appendix 3).

## Results

### Characteristics of the Included Papers

After the initial screening, we identified 4122 valid sample papers on digital health interventions. Following the second round of full-paper screening, and after excluding interventions that did not incorporate any strategies, 885 studies ultimately met the eligibility criteria.

**Figure 2:**
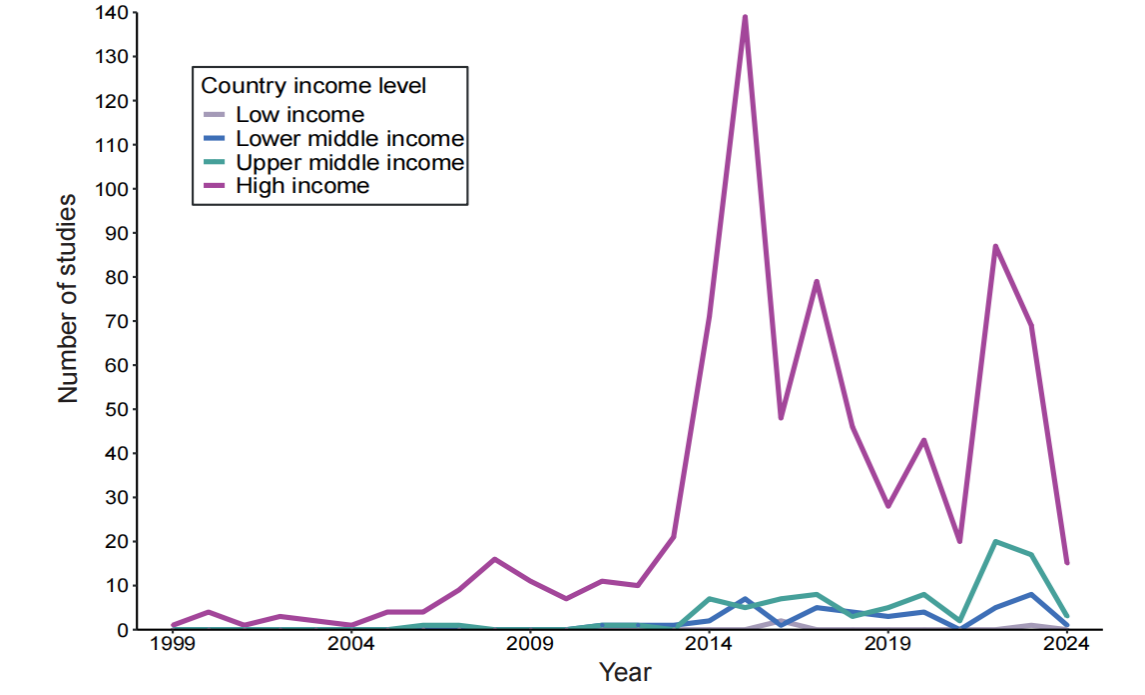
Time trends of different income-level countries in the analyzed papers.

According to the World Bank [40], countries were categorized into 4 income levels: low income, lower middle income, upper middle income, and high income (Figure 2). The number of studies conducted in high-income countries (n=750, 84.7%) far exceeded that of other income levels, with research activity increasing in all countries since 2010. Notably, studies in high-income countries peaked significantly around 2015, with 139 (15.7%) studies. Studies in upper–middle-income and lower–middle-income countries showed moderate activity with occasional peaks, particularly in 2022 (n=22, 2.5%) and 2023 (n=8, 0.9%), respectively.

The sample papers reported a total of 954,847 participants (10 papers did not report the number of participants, and we were unable to identify them), ranging from a minimum of 6 to a maximum of 333,669. Participant ages varied significantly, ranging from children as young as 18 months to adults over 80 years old. Among the sample papers, 238 (26.9%)focused on vulnerable groups: 86 (9.7%)on children and adolescents, 95 (10.7%) on women, 33 (3,7%) on older adults, and 5(0.6%) on LGBT (lesbian, gay, bisexual, and transgender) individuals. Additionally, 223 (25.2%) studies involved ethnic minorities: 5 (0.6%) on African American participants, 2 (0.2%) on Hispanic participants, 4 (0.5%) on Asian participants, 4 (0.5%) on Black participants, 2 (0.2%) on Latino or Latina populations, and 4 (0.5%) on American Indian or Alaska Native participants. The remaining studies involved other ethnic minority groups.

### Characteristics of the Intervention

**Figure 3:**
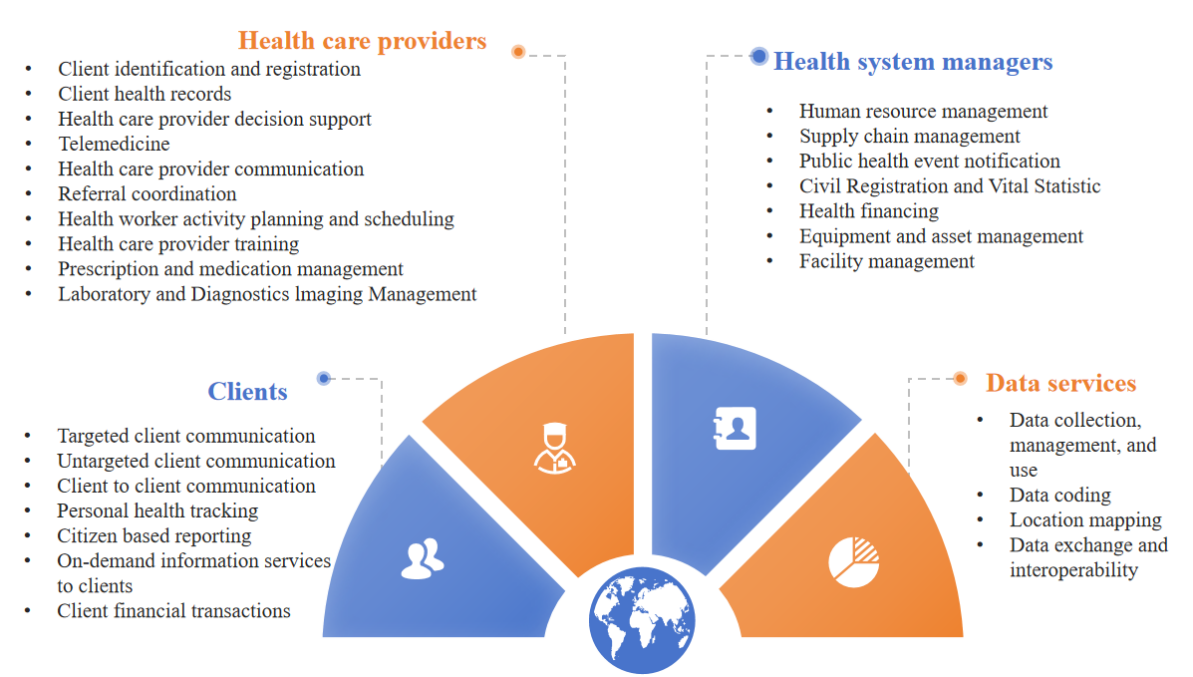
World Health Organization (WHO) classification of digital health interventions.

The WHO has categorized digital health interventions into 4 groups based on their intended users: consumers, health care providers, health system managers, and data services [39] (Figure 3). In this study, out of the total 885 interventions analyzed, the vast majority (844, 95.4%) were designed for clients. Health care providers were the target users for 62 (7%) interventions, while health system managers and data services were the focus of only 6 (0.7%) and 1 (0.1%) interventions, respectively.

We classified the intervention settings into 5 categories: community, hospital, home, school, and other. Out of the total interventions, 118 (13.3%) were identified as taking place in the community setting, 268 (30.3%) in the hospital setting, 237 (26.8%) in the home setting, 50 (5.6%) in the school setting, and 212 (24.1%) in other settings, which included locations such as companies, workplaces, and laboratories. Among the studies included, 858 (93.2%) focused on one or more specific health issues.

We identified 23 different types of delivery in the included studies and classified them into four categories: (1) phone-based, including short message service, phone calls, and interactive voice response; (2) web-based, including email, health electronic record, online telecare/training platform, forums, and interactive videos; (3) app-based, including smartphone/tablet-computer apps, videoconferencing software, and social media apps; (4) other, including wearable devices, telemonitoring device, virtual reality, artificial intelligence, and games. The most frequently used delivery type is web-based (n=282, 31.9%), followed by phone-based (n=266, 30%) and app-based (n=259, 29.3%). Of the delivery senders, 384 (43.4%) utilized automatic support, 457 (53.7%) employed human support, and 43 (4.9%) used both.

Theories and models play a crucial role in the development, implementation, and evaluation of digital health interventions by providing a framework for understanding the complex factors that influence health behaviors and guiding the selection of appropriate intervention strategies. In this study, 92 (10.4%) interventions reported using theories or models to inform their design and delivery, with some interventions using theories or models alone, while others used a combination of both. After combining the same theories or models mentioned across the included papers, a total of 13 distinct theories and 17 distinct models were identified. The most frequently used theory was Social Cognitive Theory, which was applied in 20 (2.3%) interventions. The Transtheoretical Model of Behavior Change was the most frequently used model, reported in 6 (0.7%) interventions.

### Identification and Categorization of Digital Health Intervention Strategies

#### Overview of the Identified Strategies

A total of 885 sample papers were identified as employing at least one strategy in digital health interventions. Among these papers, 173 (19.5%) distinct strategies were identified and subsequently categorized into 19 themes (Multimedia Appendix 4). Of these themes, 18 represented clearly categorizable strategies, while 1 theme, labeled as “other,” was used to group strategies that did not fit into the predefined categories.

Among the identified strategy themes (Table 1), the most frequently used was “guide,” employed in 492 (55.6%) out of the 885 papers. The second most common theme was ”monitor,” which was utilized in 490 (55.4%) papers. The third most prevalent theme was “communication,” which was applied in 392 papers, constituting 44.3% of the total. Other commonly used strategy themes included ”engagement“ (n=384, 43.3%), ”support“ (n=268, 30.3%), ”stimulation“ (n=271, 30.6%), ”management“ (n=263, 29.7%), ”feedback“ (n=236, 26.7%), ”goal setting“ (n=228, 25.8%), and ”action planning“ (n=203, 22.9%).

**Table 1:** The general results of the identified strategies.

Theme	Identified papers, n (%)
Guide	492 (55.6)
Monitoring	490 (55.4)
Communication	392 (44.3)
Engagement	384 (43.4)
Stimulate	271 (30.6)
Support	268 (30.3)
Management	263 (29.7)
Feedback	236 (26.7)
Goal setting	228 (25.8)
Action planning	203 (22.9)
Shaping	189 (21.4)
Tailor	184 (20.8)
Others	132 (14.9)
Prompts	128 (14.5)
Reward	69 (7.8)
Cues	62 (7)
Identity	57 (6.4)
Restructure	19 (2.1)
Model/demonstrate	10 (1.1)

#### Temporal, Geographic, and Demographic Developments in Digital Health Intervention Strategies

To observe how strategies have changed over time, we selected the year 2017 as the cut-off point. This is because 2017 marked a few significant milestones in digital health regulation and global health policy. In the United States, the Food and Drug Administration (FDA) launched its Digital Health Innovation Action Plan and established the Digital Health Unit [41]. Concurrently, the WHO initiated the development of its first guideline on digital health interventions [42]. These initiatives at both national and international levels potentially influenced the development of digital health intervention practice globally [43]. In this study, we found that before 2017, the top 3 strategies were ”monitoring“ (n=205, 51.4%), ”communication“ (n=198, 49.6%), and ”guide“ (n=197, 49.4%), with ”monitoring“ being the most frequently used strategy. From 2017 onward, the top 3 strategies shifted to ”guide“ (n=295, 60.8%), ”monitoring“ (n=285, 58.8%), and ”engagement“ (n=209, 43.1%), with ”guide“ becoming the most prevalent strategy. The specific details are presented in Table 2.

**Table 2:** The results of the identified strategies before and after 2017.

Before 2017		After 2017	
Theme	Value, n (%)	Theme	Value, n (%)
Monitoring	205 (51.4)	Guide	295 (60.8)
Communication	198 (49.6)	Monitoring	285 (58.8)
Guide	197 (49.4)	Engagement	209 (43.1)
Engagement	175 (43.9)	Communication	194 (40.0)
Stimulate	134 (33.6)	Support	138 (28.5)
Feedback	133 (33.3)	Stimulate	137 (28.2)
Management	131 (32.8)	Management	132 (27.2)
Support	130 (32.6)	Shaping	109 (22.5)
Goal setting	123 (30.8)	Goal setting	105 (21.6)
Action planning	112 (28.1)	Feedback	103 (21.2)
Prompts	86 (21.6)	Tailor	102 (21.0)
Tailor	82 (20.6)	Action planning	91 (18.8)
Shaping	80 (20.1)	Others	74 (15.3)
Others	58 (14.5)	Identity	46 (9.5)
Cues	34 (8.5)	Reward	46 (9.5)
Reward	23 (5.8)	Prompts	42 (8.7)
Identity	11 (2.8)	Cues	28 (5.8)
Model/demonstrate	10 (2.5)	Restructure	10 (2.1)
Restructure	9 (2.3)	Model/demonstrate	0 (0)

**Figure 4:**
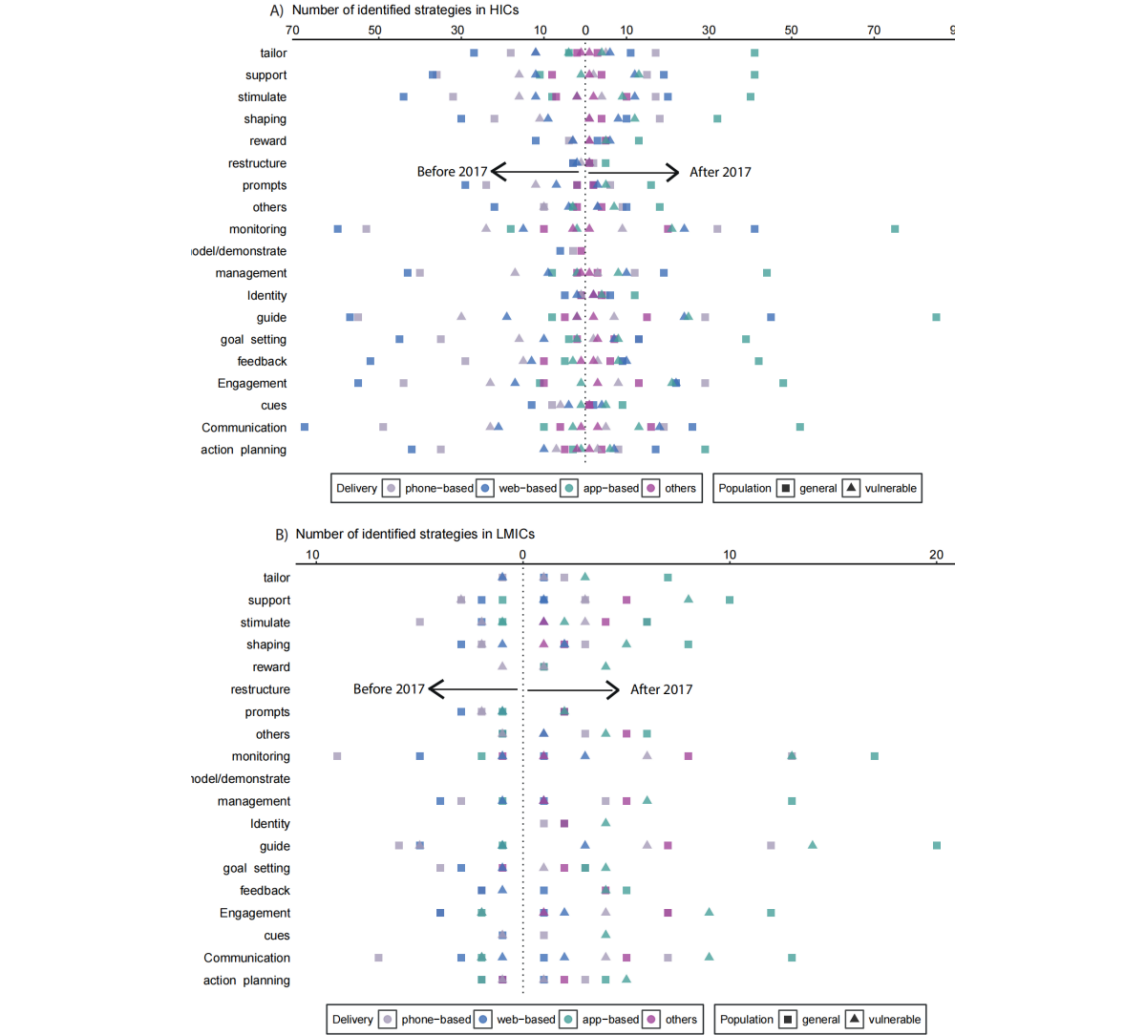
The distribution of strategies across delivery, population, and time in high-income countries (HICs) and low- and middle-income countries (LMICs).

Figure 4 shows the distribution of strategies in high-income countries and low- and middle-income countries before and after 2017, illustrating the number of identified strategies categorized by delivery type (phone-based, web-based, app-based, and other) and population type (general and vulnerable). High-income countries demonstrated a substantially higher number and diversity of strategies, with ”monitoring,” “guide,” and ”communication“ being the most prevalent. While similar strategies were prioritized in low- and middle-income countries, they were implemented with less intensity. After 2017, both high-income and low- and middle-income countries showed an increase in the number of strategies employed, with high-income countries exhibiting more pronounced growth. This development was particularly evident in the adoption of emerging strategies such as ”engagement,” “tailoring,” and “support.”

Regarding population focus, the number of studies targeting the public far exceeded those focusing on vulnerable populations (including women, children, youth, seniors, minorities, LGBT, and low-income groups), at 73.1% (n=647) and 26.9% (n=238), respectively. While both country groups showed strategies targeting general and vulnerable populations, high-income countries demonstrated a greater emphasis on interventions for vulnerable groups, especially after 2017.

#### Distribution of Digital Health Intervention Strategies Across Intervention Duration and Senders

**Figure 5:**
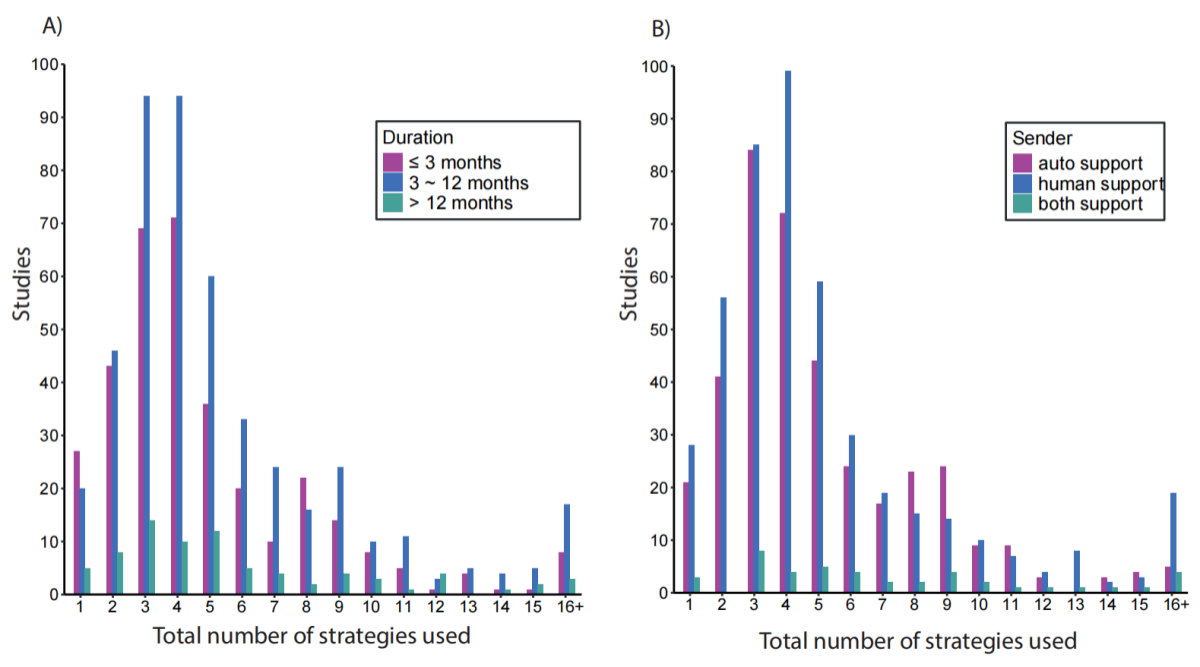
Distribution of strategies across the intervention duration and senders.

Figure 5 illustrates the distribution of strategies across intervention duration and senders. Across all duration categories, there was a clear peak between 3 and 4 strategies, with a gradual decline as the number of strategies increased. Moreover, interventions implementing more than 10 strategies were relatively uncommon. Regarding intervention duration, short-term (≤3 months) and medium-term (3-12 months) interventions dominated the landscape, particularly those employing between 2 and 4 strategies. Medium-term interventions showed the highest frequency, especially for interventions utilizing between 2 and 5 strategies. Long-term interventions (>12 months), while less common, maintained a consistent presence across the spectrum of strategy numbers.

The distribution across sender types demonstrated that human support was the most prevalent delivery form, particularly for those using between 2 and 5 strategies. Automated support followed a similar pattern but with lower frequencies, while combined support (both human and automated) was the least common but had a consistent presence. According to the duration graph, all sender types peaked at 3 to 4 strategies used, with a sharp decline in frequency as the number of strategies increased beyond 5.

## Discussion

### Principal Findings

Our study provided a comprehensive analysis of strategies employed in digital health interventions over the past 25 years. We identified 173 unique strategies and categorized them into 19 overarching themes based on BCTs and BSs. This helped us establish a valuable framework for understanding the diverse approaches used in designing and implementing digital health interventions. The analysis revealed significant insights into several key aspects—the evolution of strategies over time, the differential involvement between high-income and low- and middle-income countries, and the influence of various factors such as delivery methods, target populations, intervention duration, and intervention providers. These findings collectively map the landscape of digital health intervention strategies across a quarter-century, providing valuable guidance for researchers and practitioners.

Specifically, we identified that the three most prevalent strategies were ”guide,“ ”monitoring,“ and ”communication,” highlighting the importance of providing clear guidance, tracking progress, and facilitating interactive communication in digital health interventions. The widespread application of “engagement” and “support” underscored the significance of fostering user involvement and providing adequate assistance throughout the intervention process. These findings were consistent with prior research that underscored the crucial role of user engagement, self-monitoring, and social support in digital health interventions [21,44].

The number of strategies used in each paper varied widely, from a single strategy to a comprehensive approach involving up to 32 strategies, highlighting the complexity and diversity of digital health interventions. Most studies fell into the low or medium categories, indicating that most digital health interventions had a focused approach with a limited set of strategies. The existing research indicated that using a combination of strategies, rather than a single approach, can be more effective in enhancing participant retention [32]. The available evidence suggests that to optimize participant retention throughout a study, researchers should consider implementing multiple retention strategies across various themes [33]. Future research should carefully evaluate the optimal strategic approach for each intervention, considering its specific goals, target population, and context. It should also investigate how the number and type of strategies employed affect intervention effectiveness and identify the best combination of strategies for various intervention contexts.

The landscape of digital health intervention strategies has undergone significant shifts, particularly around the pivotal year of 2017. This year marked 2 crucial developments: the launch of the Digital Health Unit by the FDA in the United States and the initiation of the WHO’s first guideline on digital health interventions. Prior to 2017, “monitoring” was the predominant strategy in digital health interventions. However, in 2017 and beyond, “guide” emerged as the most frequently employed approach. This transition reflected a shift toward more dynamic and interactive user interfaces, emphasizing proactive guidance rather than passive monitoring [45]. This change aligned with emerging evidence suggesting that interactive and personalized digital health interventions can lead to better user engagement and health outcomes [46,47]. Additionally, “engagement” had also risen in importance after 2017, illustrating a growing focus on enhancing user experience and satisfaction to improve intervention outcomes [48]. This change underscored a broader trend within the health care sector toward patient-centered care, which prioritizes the user experience and active participation in health management [49].

The adoption of strategies in digital health interventions was closely related to delivery methods and sender types [50]. While web-based and phone-based interventions remained prevalent, the rise of app-based strategies reflected the increasing penetration of smartphones, aligning with the ubiquitous use of mobile phones in modern society and highlighting the potential of leveraging mobile technology for health interventions [51]. This trend aligned with the ubiquitous use of mobile devices and underscored the growing importance of accessible, on-the-go health solutions. Regarding sender types, most of the sample papers employed human support in the delivery of digital health interventions, while less than half used automatic support. This distribution highlighted the ongoing significant role of human involvement in these interventions. Despite the increasing capabilities of automated systems, human support remained a crucial component in many digital health interventions, attributed to the human ability to provide empathy, build rapport, and tailor interventions to individual needs. Nonetheless, the role of automatic support was expanding, driven by advances in artificial intelligence and its potential for scalability and cost-effectiveness [52]. This dual trend—the continued importance of human support alongside the growth of automated systems—suggested a future where digital health interventions may increasingly leverage both human and artificial intelligence to improve health outcomes.

There was a distinct regional bias in the use of strategies, particularly across different income levels. High-income countries demonstrated a substantially higher number and diversity of identified strategies compared to low- and middle-income countries. This disparity may be attributed to better technological infrastructure, greater financial resources, and higher digital literacy among the population in high-income countries [50]. While low- and middle-income countries showed lower overall usage, they displayed a growing trend in adopting digital health strategies after 2017. This rise indicated a catching-up phenomenon, possibly driven by increased global health initiatives, international collaborations, and high mobile technology penetration in many low- and middle-income countries [53].

Regarding population distribution, the results indicated that the number of studies targeting the public far exceeded those focusing on vulnerable populations, suggesting a potential gap in addressing the specific needs of vulnerable groups, including women, children, youth, seniors, minorities, LGBT, and low-income populations, through digital health interventions [54]. Future research and implementation efforts should focus on developing more targeted strategies for these underserved populations to ensure equitable access to digital health benefits.

In conclusion, over the past 25 years, digital health interventions and their strategies have undergone significant developments. Initially, digital health interventions often utilized relatively simple and limited strategies, mainly focusing on providing information and basic self-monitoring functions [55]. With advancements in technology and a deeper understanding of behavior change mechanisms, digital health intervention strategies have become increasingly complex and diverse. Recently, there has been a rise in the use of personalized and adaptive strategies, aiming to tailor interventions to participants' characteristics, needs, and responses [56], as well as the aims of the intervention. Personalized or individualized strategies have become increasingly popular and widely used due to their potential to enhance engagement and intervention effectiveness. Notably, there has been a rise in the use of transtheoretical strategies in recent years, integrating elements from various behavior change theories to create more comprehensive and effective interventions [57].

### Strengths of Our Research

Our findings built upon and broadened the scope of previous research on digital health intervention strategies [20,22,26-34,58] and the principles strategy classification [22,35]. While previous studies have often concentrated on specific health domains or populations, our research offered a more comprehensive view of the strategies used across various health issues and target groups. By identifying and categorizing a wider array of strategies, we provided a more integrated understanding of how to promote health behavior change and enhance health outcomes in the digital era.

Additionally, our analysis of the diversity in the number of strategies employed in each paper highlights the complexity and variability inherent in digital health interventions. Although prior research has underlined the significance of employing multiple strategies to tackle the multifaceted nature of health behavior change, our findings suggested that the ideal number of strategies might differ based on the specific objectives and context of the intervention. This emphasized the necessity for a customized approach in the design and implementation of digital health interventions, considering the distinct needs and characteristics of the target population and health domain.

### Implications for Research and Practice

The findings carried several crucial implications for research and practice in the digital health field. First, by amalgamating evidence on what strategies prove effective across diverse health domains and populations, our study presented a detailed categorization of strategies for researchers and practitioners aiming to design and evaluate digital health interventions. This structured framework facilitated an understanding of the varied approaches within these interventions, aiding in the strategic selection and combination tailored to specific intervention goals and contexts. This, in turn, aimed to maximize the efficacy and impact of digital health interventions.

Second, this analysis of the frequency and distribution of strategy themes can direct future research priorities and resource allocation. The prominent presence of strategies concerning guidance, monitoring, and communication underlines their potential significance as focal areas for additional research.

Third, the observed variation in the number of strategies employed across different studies underscored the necessity for a more refined approach to designing and assessing digital health interventions. It suggested moving away from the presumption that employing more strategies invariably leads to better outcomes. Instead, there is a need for a deliberate consideration of the most effective number and mix of strategies, aligning with the unique requirements and attributes of the target population and health domain.

### Limitations of This Study

This study has several limitations. Despite following a comprehensive search strategy, some relevant and important papers may have been overlooked, especially those published in languages other than English, from low- and middle-income countries, and in gray literature due to the limitations of the databases chosen. The process of data extraction and categorization of strategies was contingent on the information provided in the sample papers, which in some instances, may have been limited or inconsistent. Moreover, the diversity among the sample papers regarding populations, health domains, and intervention designs might restrict the applicability of our findings to specific situations. Additionally, this study did not carry out a comparative analysis of the effects of different strategies, nor did it explore and quantify the relationship between strategies and health outcomes.

### Conclusions

This study provided an exhaustive synthesis of strategies used in digital health interventions over the past 25 years, highlighting their characteristics, distribution, and diversity in promoting health behavior changes and improving outcomes. By identifying and categorizing 173 unique strategies within 19 overarching themes, this study offered a comprehensive framework for understanding the complex approaches used in digital health intervention strategies. Additionally, this study highlighted that high-income and low- and middle-income countries have seen increased adoption after 2017, and human support remained crucial despite advancements in automated systems. These findings facilitated the careful design and assessment of future digital health interventions, significantly contributing to the establishment of evidence-based guidelines and best practices that enhance their efficacy.

## Appendix

Multimedia Appendix 1 PRISMA-ScR (Preferred Reporting Items for Systematic Reviews and Meta-Analyses extension for Scoping Reviews) checklist.

Multimedia Appendix 2 Search strategy.

Multimedia Appendix 3 Framework for Strategy Identification and Definition.

Multimedia Appendix 4 Overview of the identified strategies.

## Abbreviations

BCT: Behavior Change Technique
BS: Behavior Strategy
FDA: Food and Drug Administration
LGBT: lesbian, gay, bisexual, and transgender
PRISMA-ScR: Preferred Reporting Items for Systematic Reviews and Meta-Analyses extension for Scoping Reviews
RCT: randomized controlled trial
WHO: World Health Organization
