# Supplementary material for: Mapping the Landscape of Digital Health Intervention Strategies: 25-Year Synthesis
Source: J Med Internet Res. 2025 Jan 13;27:e59027. doi: 10.2196/59027 (PMC11773286; doi:10.2196/59027)
Supplement: Multimedia Appendix 1 [file jmir_v27i1e59027_app1.pdf]

Section/topic	#	Checklist item	Location(s) Reported
**INFORMATION SOURCES AND METHODS**			
Database name	1	Name each individual database searched, stating the platform for each.	PubMed, Web of Science, and Scopus
Multi-database searching	2	If databases were searched simultaneously on a single platform, state the name of the platform, listing all of the databases searched.	N/A
Study registries	3	List any study registries searched.	N/A
Online resources and browsing	4	Describe any online or print source purposefully searched or browsed (e.g., tables of contents, print conference proceedings, web sites), and how this was done.	N/A
Citation searching	5	Indicate whether cited references or citing references were examined, and describe any methods used for locating cited/citing references (e.g., browsing reference lists, using a citation index, setting up email alerts for references citing included studies).	N/A
Contacts	6	Indicate whether additional studies or data were sought by contacting authors, experts, manufacturers, or others.	NO
Other methods	7	Describe any additional information sources or search methods used.	N/A
**SEARCH STRATEGIES**			
Full search strategies	8	Include the search strategies for each database and information source, copied and pasted exactly as run.	"digital health" OR "digital intervention" OR dhealth OR d-health OR ehealth OR "electronic health" OR e-health OR mhealth OR "mobile health" OR m-health OR telehealth OR teletherapy OR tele-therapy OR tele-intervention OR teleintervention OR telecare OR tele-care OR "wearable device" OR wearables OR telemedicine (Topic) and 1999-2024 (Year Published) and "randomized controlled trial" OR rct OR "cluster randomized controlled trials" OR "cluster RCT" (Topic) and English (Language) and Article (Document Types) and Article (Document Types) and Article (Document Types) and Article (Document Types) and Article (Document Types)<br>(("digital health"[Title/Abstract] OR "digital intervention"[Title/Abstract] OR "dhealth"[Title/Abstract] OR "d-health"[Title/Abstract] OR "ehealth"[Title/Abstract] OR "electronic health"[Title/Abstract] OR "e-health"[Title/Abstract] OR "mhealth"[Title/Abstract] OR "mobile health"[Title/Abstract] OR "m-health"[Title/Abstract] OR "telehealth"[Title/Abstract] OR "teletherapy"[Title/Abstract] OR "tele-therapy"[Title/Abstract] OR "tele-intervention"[Title/Abstract] OR "teleintervention"[Title/Abstract] OR "telecare"[Title/Abstract] OR "tele-care"[Title/Abstract] OR "wearable device"[Title/Abstract] OR "wearables"[Title/Abstract] OR "telemedicine"[Title/Abstract] OR "electronic health record"[Title/Abstract] OR "EHR"[Title/Abstract] OR "electronic medical record"[Title/Abstract] OR "EMR"[Title/Abstract] OR "information communication technology"[Title/Abstract] OR "ICT"[Title/Abstract] OR "telemonitoring"[Title/Abstract] OR "social media"[Title/Abstract] OR "digital media"[Title/Abstract] OR "electronic media"[Title/Abstract] OR "app"[Title/Abstract] OR "mobile application"[Title/Abstract] OR "phone"[Title/Abstract] OR "short-message service"[Title/Abstract] OR
Limits and restrictions	9	Specify that no limits were used, or describe any limits or restrictions applied to a search (e.g., date or time period, language, study design) and provide justification for their use.	Time range: T January 1, 1999, to March 10, 2024;<br>Language restriction: English;<br>Study design restriction: randomized controlled trials (RCTs);

Search filters	10	Indicate whether published search filters were used (as originally designed or modified), and if so, cite the filter(s) used.	We used a combination of built-in search filters and custom search limits in our literature search across multiple databases. The specific filters and limits applied to each database are as follows:<br>1.PubMed:<br>1.Text availability: Full text (applied using the 'Text availability' filter)<br>2.Article type: Not Review or Systematic Review or Meta-Analysis (applied using the built-in 'Article type' filter)<br>3.Custom date range: 1 January 1999 to 10 March 2024 (applied using the 'Custom range' feature)<br>2.Web of Science:<br>1.Publication year: 1999-2023 (applied using the 'Year Published' filter)<br>2.Document Type: Article (applied using the 'Document Types' filter)<br>3.Scopus:<br>1.Year: 1999-2024 (applied using the 'Year' filter)<br>2.Article type: Article (applied using the 'Document Type' filter)<br>3.Language: English (applied using the 'Language' filter)
Prior work	11	Indicate when search strategies from other literature reviews were adapted or reused for a substantive part or all of the search, citing the previous review(s).	N/A
Updates	12	Report the methods used to update the search(es) (e.g., rerunning searches, email alerts).	N/A
Dates of searches	13	For each search strategy, provide the date when the last search occurred.	15-Mar-24
**PEER REVIEW**			
Peer review	14	Describe any search peer review process.	We conducted peer review of the search strategy at two points during the literature search process to ensure the comprehensiveness, accuracy, and reproducibility of the search strategy.<br><br>The first peer review was performed before running the searches. Three researchers with expertise in systematic reviews and literature searching assessed the search strategy. The review included an evaluation of the search terms, database selection, and the use of filters for each database. Based on the feedback from the peer review, we modified the search strategy, including the addition of relevant synonyms and subject headings, and the removal of redundant or irrelevant search terms.<br><br>The second peer review was conducted after the initial searches were completed. The same researcher reviewed the modified search strategy once again and assessed the relevance of the search results. Based on the feedback from the second peer review, we further refined the search strategy to ensure the comprehensiveness and accuracy of the search.<br><br>Through these two rounds of peer review, our search strategy was significantly improved, resulting in a comprehensive, accurate, and reproducible search protocol.
**MANAGING RECORDS**			
Total Records	15	Document the total number of records identified from each database and other information sources.	**12,372**

Deduplication	16	Describe the processes and any software used to deduplicate records from multiple database searches and other information sources.	We used Microsoft Excel software to deduplicate records from multiple database searches and other information sources.<br>The specific steps were as follows:<br>1.All search results from databases and other information sources were exported and saved in Excel format.<br>2.All Excel files were combined into one master Excel workbook, with each database or information source occupying a separate worksheet.<br>3.A new worksheet named "Deduplicated Records" was created in the master workbook.<br>4.The records from each database or information source worksheet were copied into the "Deduplicated Records" worksheet.<br>5.Excel's "Data" - "Remove Duplicates" function was used, selecting key fields such as "Author," "Title," "Journal," "Volume," "Issue," and "Publication Year" as the basis for identifying duplicates.<br>6.The duplicate records identified by Excel were carefully checked to ensure that they were indeed duplicates. In rare cases, different databases may use slightly different title or author name formats for the same article, requiring manual verification.<br>7.Confirmed duplicate records were removed, keeping a single unique record.<br>8.The number of records before and after deduplication was recorded for reporting purposes.
