# Supplementary material for: Mapping the Landscape of Digital Health Intervention Strategies: 25-Year Synthesis
Source: J Med Internet Res. 2025 Jan 13;27:e59027. doi: 10.2196/59027 (PMC11773286; doi:10.2196/59027)
Supplement: Multimedia Appendix 2 [file jmir_v27i1e59027_app2.docx]

Appendix 2. Search Strategy

**PubMed**

| **"digital health" OR “digital intervention” OR dhealth OR d-health OR ehealth OR "electronic health" OR e-health OR mhealth OR "mobile health" OR m-health OR telehealth OR teletherapy OR tele-therapy OR tele-intervention OR teleintervention OR telecare OR tele-care OR “wearable device” OR wearables OR telemedicine** (Topic) and **1999-2024** (Year Published) and **"randomized controlled trial" OR rct OR "cluster randomized controlled trials" OR "cluster RCT"** (Topic) and **English** (Language) and **Article** (Document Types) and **Article** (Document Types) and **Article** (Document Types) and **Article** (Document Types) and **Article** (Document Types) |
| --- |

| (("digital health"[Title/Abstract] OR "digital intervention"[Title/Abstract] OR "dhealth"[Title/Abstract] OR "d-health"[Title/Abstract] OR "ehealth"[Title/Abstract] OR "electronic health"[Title/Abstract] OR "e-health"[Title/Abstract] OR "mhealth"[Title/Abstract] OR "mobile health"[Title/Abstract] OR "m-health"[Title/Abstract] OR "telehealth"[Title/Abstract] OR "teletherapy"[Title/Abstract] OR "tele-therapy"[Title/Abstract] OR "tele-intervention"[Title/Abstract] OR "teleintervention"[Title/Abstract] OR "telecare"[Title/Abstract] OR "tele-care"[Title/Abstract] OR "wearable device"[Title/Abstract] OR "wearables"[Title/Abstract] OR "telemedicine"[Title/Abstract] OR "electronic health record"[Title/Abstract] OR "EHR"[Title/Abstract] OR "electronic medical record"[Title/Abstract] OR "EMR"[Title/Abstract] OR "information communication technology"[Title/Abstract] OR "ICT"[Title/Abstract] OR "telemonitoring"[Title/Abstract] OR "social media"[Title/Abstract] OR "digital media"[Title/Abstract] OR "electronic media"[Title/Abstract] OR "app"[Title/Abstract] OR "mobile application"[Title/Abstract] OR "phone"[Title/Abstract] OR "short-message service"[Title/Abstract] OR "sms"[Title/Abstract] OR "text-message"[Title/Abstract] OR "virtual reality"[Title/Abstract] OR "blogging"[Title/Abstract] OR "e-mail"[Title/Abstract] OR "email"[Title/Abstract] OR "electronic mail"[Title/Abstract]) AND ("randomized controlled trial"[Title/Abstract] OR "RCT"[Title/Abstract] OR "cluster randomized controlled trial"[Title/Abstract] OR "cluster RCT"[Title/Abstract]) AND "randomized controlled trial"[Publication Type] AND 1999/01/01:2025/01/01[Date - Publication] AND "English"[Language]) |
| --- |

**Web of Science**

| ( TITLE-ABS-KEY ( "digital health" OR "digital intervention" OR dhealth OR d-health OR ehealth OR "electronic health" OR e-health OR mhealth OR "mobile health" OR m-health OR telehealth OR teletherapy OR tele-therapy OR tele-intervention OR teleintervention OR telecare OR tele-care OR "wearable device" OR wearables OR telemedicine ) AND TITLE-ABS-KEY ( "randomized controlled trial" OR "RCT" OR "cluster randomized controlled trial" OR "cluster RCT" ) AND LANGUAGE ( english ) ) AND PUBYEAR > 1998 AND PUBYEAR < 2025 AND ( LIMIT-TO ( DOCTYPE , "ar" ) ) AND ( LIMIT-TO ( EXACTKEYWORD , "Randomized Controlled Trial" ) ) |
| --- |

**Scopus**
