# Supplementary material for: Mapping the Landscape of Digital Health Intervention Strategies: 25-Year Synthesis
Source: J Med Internet Res. 2025 Jan 13;27:e59027. doi: 10.2196/59027 (PMC11773286; doi:10.2196/59027)
Supplement: Multimedia Appendix 3 [file jmir_v27i1e59027_app3.doc]

Appendix 3. The define and framework for identifying strategies

| **Theme** | **Items** | **Description** |
| --- | --- | --- |
| **Action Planning** | Action planning (including implementation intentions) | Entail comprehensive strategizing regarding an individual's actions, encompassing at least timing, context, location and/or situation of take actions. Plans that individuals make about when, where, and how to act in a particular way to achieve a goal. |
|  | Coping planning | Think ahead about potential obstacles that might hinder goal achievement and devising strategies to overcome them. |
|  | Plan social change | Strategize actions to facilitate or implement change on a societal level. |
|  |  |  |
| **Communication** | General communication skills training | This refers to a complex array of communication strategies that exploit mass media's expansive reach, social mobilization's vigor, and community leaders' influence. |
|  | Person-to-machine communication | Allude to the engagement between individuals and automated systems or devices, empowering users to communicate information, commands, and requests with machines across various facets of intervention delivery. |
|  | Person-to-person communication | Support the efficient communication of messages at the interpersonal level to aid in achieving objectives. |
|  | Self-talk | The practice of individuals engaging in self-dialogue, either vocally or internally, prior to and while executing predetermined actions, in order to ensure continuity in activities. |
|  | Counseling | Involve “educating patients” or “feedback, self-monitoring, and reinforcement”. |
|  | Visit | Attend to participants throughout the intervention development process to determine their perceived role and anticipated extent of engagement in various aspects of intervention delivery. |
|  |  |  |
| **Cues** | Self-referent cues | Cues can be used to remind individuals to engage in a particular behavior or an alternative behavior. Contain explicit personally relevant information (e.g. user’s name), effectively influencing individuals to exhibit the desired behavior. |
|  | Discriminative (learned) cues | Provide technological tools like mobile phone notifications that influence individuals to execute the prescribed behavior. |
|  |  |  |
| **Engagement** | Engage | Represent the quantity, frequency, duration, and depth of use, coupled with a subjective evaluation involving attentiveness, interest, and emotional response. |
|  | Follow up | An evolving interaction, anticipated to fluctuate amongst individuals and within an individual over time. |
|  | Discuss | Stimulate individuals to participate in discussions that foster a dialogue conducive to change, aimed at mitigating resistance and addressing ambivalence toward change. |
|  | Interview | Driven by the necessity to enhance coherence and sense-making, interviews are scheduled between participants and the research team to offer insights on DHIs to equip participants with the knowledge needed to facilitate the adoption of DHIs. |
|  |  |  |
| **Feedback** | Feedback | Involve offering participants individualized feedback on their recorded behaviors, evaluations of service performance, and promoting constructive reflexive monitoring practices, all aimed at improving overall engagement with the interventions. |
|  | Biofeedback | Information regarding the biostatistics of patients registered at each healthcare facility is gathered. |
|  |  |  |
| **Goal Setting** | Behavioral resolution | The participants are motivated to make a behavioural resolution, with a focus on either modifying or maintaining particular behaviors (e.g. take more exercise in the upcoming time and lose/maintain weight) |
|  | Set graded tasks | Deconstructing the desired behavior into smaller, more manageable tasks and empowering individuals to progress from minor accomplishments towards achieving the ultimate behavior goal. Regularly reviewing behavioral and outcome goals. |
|  | Outcome goal(s) | The goal could result from one or multiple behaviors, yet it is not a behavior in itself. |
|  |  |  |
| **Guide** | Learn | Participants are provided with educational materials enabling their involvement in the process of DHIs. |
|  | Provide instruction | Summaries of the intervention components and instructional materials are created and distributed to participants during training sessions to assist them in navigating the process of DHIs. |
|  | Recommendation | Participants are advised on the characteristics of the DHIs, including its evidence base, theoretical framework, participatory design, and the potential benefits for participants, to align with their particular practices. |
|  | Regulate negative emotions | Encourage cognitive engagement by highlighting the advantages for participants and practices (coherence) throughout the guide training sessions.Enhance participants' confidence in utilizing DHIs and alleviate any distrust, anxiety, resistance, or other negative emotions stemming from uncertainty. |
|  |  |  |
| **Identity** | Identification myself | Participants are navigating a health issue that involves identification of self as role model, self-affirmation, identity linked to altered behavior, reframing, or cognitive dissonance. |
|  | Barrier identification | Participants are encouraged to reflect on potential obstacles and strategize ways to overcome them. Barriers could encompass behavioral, cognitive, emotional, environmental, social, and/or physical challenges. |
|  |  |  |
| **Management** | Self-management | Afford the participants the ability to select their goals from a predetermined inventory, supporting their autonomy and facilitating accommodation for the individuals’ evolving needs and personal advancement through the DHIs process. |
|  | Emotional management | A set of specific approaches indirectly focuses on behavior change by reducing anxiety and stress, employing tactics to decrease negative emotions and regulate mood to facilitate desired behavior, and enhance positive emotions for improved behavioral performance. |
|  | Automated management | This refers to any technique designed to assist individuals in regulating their behavior automatically. These methods do not directly address the performance of the target behavior but aim to simplify manual operation when it is necessary. |
|  |  |  |
| **Model/Demonstrate** | Modeling | Involves instructing individuals on behavior execution, such as through physical or visual demonstrations, either in-person or remotely.Through "demonstrations," individuals can observe the behavior being enacted, commonly seen when providing instructions on behavior performance. |
|  |  |  |
| **Monitoring** | Monitoring of outcome of behaviour | The participant is requested to maintain a log of defined metrics that are anticipated to be impacted by the behaviour change (e.g., blood pressure, blood glucose levels, weight loss, physical fitness). |
|  | Self-monitoring | Participant is asked with documenting specific behaviors as a strategy for behavior change (e.g., take the form of a diary or completing behavior-related questionnaires detailing type, frequency, duration, and/or intensity). It should be clearly outlined as an intervention component, distinct from completing measures for research purposes. |
|  | Automatic monitoring | Maintain records related to the process of DHIs automatically as a designated intervention component. |
|  | Reminder | Notifications, messages, and/or alerts are dispatched by the research team to participants as reminders for DHIs and to incentivize them to maintain specific behaviors. |
|  |  |  |
| **Prompts** | Prompting generalisation of behavior | Following the demonstration of the behavior in a specific situation, individuals are motivated or supported to practice it in a diverse range of settings. Aim to detach the behavior from a single situation and integrate its application across various times and contexts. |
|  | Prompting focus on past success | Involves instructing individuals to reflect upon or document past successes in executing the behavior that occurred prior to the intervention. |
|  | Use of follow-up prompts | Refer to a process where intervention components decrease in intensity, duration, and frequency as time progresses (e.g. shifting from face-to-face interactions to methods like letters or telephone calls and/or extending the time intervals between interventions). |
|  | Prompt use of imagery | Teach individuals to visualize successfully executing the behavior or envisioning it as effortless, including breaking down the behavior into its components or simpler versions. |
|  | Prompt self talk | Motivate individuals to engage in self-talk (whether aloud or internally) prior to and during planned behaviors to enhance, reinforce, and sustain their actions. |
|  | Prompt anticipated regret | Involves creating anticipations of future regret regarding the execution or omission of a behavior. It emphasizes how individuals will emotionally respond in the future, particularly whether they will experience regret or remorse for not choosing a different course of action. |
|  | Prompt practice/rehearsal | Involve individuals repeatedly performing desired behaviors as a method employed in interventions. Aim to improve the acquisition, mastery, retention, and application of target behaviors to improve performance. |
|  |  |  |
| **Restructure** | Restructuring the physical environment | Participants are motivated to adapt the physical environment to better facilitate the target behaviourr, including modifying cues or reinforcements (e.g. disposing of high-calorie snacks or bringing their running shoes to work). |
|  | Restructuring the social environment | Participants are encouraged to adjust the social environment to enhance support for the desired behavior, which involves modifying cues or reinforcements (for example, suggesting friends engage in exercise together instead of consuming alcohol). |
|  | Avoidance/Changing exposure to cues for the behavior | Participants are encouraged to steer clear of or modify their exposure to reduce the likelihood of engaging in maladaptive behaviors. Involve identifying triggers or stimuli that precipitate the undesirable behavior and then deliberately avoiding them, or altering one's environment to minimize contact with those cues(e.g. participants may be advised to steer clear of environments where smoking occurs as part of a smoking cessation effort). |
|  |  |  |
| **Reward** | Social reward |  |
|  | Material reward |  |
|  | Self reward |  |
|  | Provide rewards for behavior | Provide timely rewards based on the effort exerted or progress made towards the target behavior. The reward or incentive can be clearly tied to the accomplishment of the particular target behavior, meaning that the individual receives the reward only upon executing the specified behavior and not receiving it if the behavior is not performed. |
|  | Other reward |  |
|  |  |  |
| **Shaping** | Habit formation | Encourage participants to develop habits or routines. |
|  | Graded tasks | At the outset, contingent rewards are provided for any progress made towards the target behavior, with an escalating level of criteria needed for the use of contingent rewards as time progresses. |
|  | Behavioral rehearsal/practice | Refer to the process of repeatedly engaging in a behavior or activity to enhance the achievement of the targeted behavior. |
|  | Habit reversal | Involve identifying and replacing unwanted habits or repetitive behaviors with more adaptive and intentional actions. Typically include raising awareness of the habit and practicing alternative responses to effectively eliminate or reduce the habit. |
|  | Reinforcing effort toward behavior | Involve a management approach of behavioral shaping that aims to enhance and reinforce an individual's commitment to the target behavior by recognizing their persistent efforts. |
|  | Shaping knowledge | Involve the process of gradually molding person's understanding, skills, or expertise through interventions. |
|  |  |  |
| **Stimulate** | Emotional stimulation | Generate optimistic expectations regarding the outcomes of DHIs, which may encompass methods aimed at enhancing positive emotions conducive to the enactment of the behavior. |
|  | Encouragement |  |
|  | Motivation |  |
|  | Threat |  |
|  | Boost self-efficacy | Enhance the conviction in personal efficacy and frame it in an affirmative manner, focusing on the immediate favorable impact of behavioral transformation rather than on the adverse repercussions of failing to amend behaviors. |
|  |  |  |
| **Support** | Practical support | Participants are furnished with tangible support in the form of resources, equipment, or opportunities that facilitate their engagement in the process of DHIs. |
|  | Social support | Involve promoting individuals to solicit social assistance to facilitate the attainment of ttarget behaviour/outcome. This incorporates support mechanisms within interventions, extending post-intervention support from intervention providers, partners, friends, and family members. |
|  | Emotional support | Observe the emotional variations of participants during the implementation of DHIs and encourage the cultivation of positive emotions to support the engagement in the targeted behavior. |
|  |  |  |
| **Tailor** | Tailor | Tailored configurations in DHIs are administered to individuals or particular groups based on the distinctive attributes of each person, encompassing demographics, clinical history, behavioral tendencies, and psychological traits. |
|  |  |  |
| **Others** |  | Other categories that do not encompass the strategy items mentioned above. |
