# Supplementary material for: Mapping the Landscape of Digital Health Intervention Strategies: 25-Year Synthesis
Source: J Med Internet Res. 2025 Jan 13;27:e59027. doi: 10.2196/59027 (PMC11773286; doi:10.2196/59027)
Supplement: Multimedia Appendix 4 [file jmir_v27i1e59027_app4.docx]

Appendix 4. Overview of identified strategies

| **Theme** | **Items** |
| --- | --- |
| **Action Planning** | Action planning (including implementation intentions) |
|  | Coping planning |
|  | Plan social change |
|  |  |
| **Communication** | General communication skills training |
|  | Person-to-machine communication |
|  | Person-to-person communication |
|  | Self-talk |
|  | Counseling |
|  | Visit |
|  |  |
| **Cues** | Self-referent cues |
|  | Discriminative (learned) cues |
|  |  |
| **Engagement** | Engage |
|  | Follow up |
|  | Discuss |
|  | Interview |
|  |  |
| **Feedback** | Feedback |
|  | Biofeedback |
|  |  |
| **Goal Setting** | Behavioral resolution |
|  | Set graded tasks |
|  | Outcome goal(s) |
|  |  |
| **Guide** | Learn |
|  | Provide instruction |
|  | Recommendation |
|  | Regulate negative emotions |
|  |  |
| **Identity** | Identification myself |
|  | Barrier identification |
|  |  |
| **Management** | Self-management |
|  | Emotional management |
|  | Automated management |
|  |  |
| **Model/Demonstrate** | Modeling |
|  |  |
| **Monitoring** | Monitoring of outcome of behaviour |
|  | Self-monitoring |
|  | Automatic monitoring |
|  | Reminder |
|  |  |
| **Prompts** | Prompting generalisation of behavior |
|  | Prompting focus on past success |
|  | Use of follow-up prompts |
|  | Prompt use of imagery |
|  | Prompt self talk |
|  | Prompt anticipated regret |
|  | Prompt practice/rehearsal |
|  |  |
| **Restructure** | Restructuring the physical environment |
|  | Restructuring the social environment |
|  | Avoidance/Changing exposure to cues for the behavior |
|  |  |
| **Reward** | Social reward |
|  | Material reward |
|  | Self reward |
|  | Provide rewards for behavior |
|  | Other reward |
|  |  |
| **Shaping** | Habit formation |
|  | Graded tasks |
|  | Behavioral rehearsal/practice |
|  | Habit reversal |
|  | Reinforcing effort toward behavior |
|  | Shaping knowledge |
|  |  |
| **Stimulate** | Emotional stimulation |
|  | Encouragement |
|  | Motivation |
|  | Threat |
|  | Boost self-efficacy |
|  |  |
| **Support** | Practical support |
|  | Social support |
|  | Emotional support |
|  |  |
| **Tailor** | Tailor |
| **Others** | Here's the revised list with prepositions in lowercase:  Automated Dialogue Therapy, Pros and Cons Analysis, Success Story Sharing, Structured Problem-Solving, Biopsychoeducation, Relaxation Training, Cognitive Approach to Stress Management, Thought Records, Catastrophic Thinking Identification, Exposure Therapy, Behavioral Experiments, Cultural Tailoring, Language Concordance, Individualized Assessment, Skills Enhancement, Access to Resources, Continuity of Care, Simple Recording System, Professional Monitoring Awareness, Multidisciplinary Team Support, Outcome Expectancy, Perceived Environmental Factors, Social Modeling, Vicarious Learning, Increased Website Exposure, Self-Regulation, Structured Self-Guided Intervention, Reframing Maneuvers, Self-Esteem Enhancement, Support Roles Assignment, Lifestyle Goals Identification, Ambivalence Resolution, Efficacy Enhancement, Discussion Forum, Group Support Sessions, Community Resource Linking, Adaptive Strategies, Self-Help Books, Web-Based Self-Disclosure Support, Low-Cost Strategies, Attribution Training, Satisfaction Assessment, Medication Image Display, Sharing Functionality, Asynchronous Therapeutic Support, Modular Program, Semi-Structured Interviews, Patient Empowerment, Decision Support, Personalized Health Information, Automated Self-Monitoring, Quality Improvement Strategies, Non-Physician Care Coordination, Motivational Enhancement, Phone-Based Group Support, Nondirective Supportive Therapy, Structured Group Education, Structured Follow-Up, Self-Guided Stress Management, Shared Decision-Making, Structured Medication List, Interactive Patient Support Tool, Longitudinal Interval Follow-Up, Self-Determination Theory Application, Standardized Protocol, Behavioral Activation, Unhelpful Pattern Changing, Behavioral Improvement Maintenance, Diary Keeping, Ongoing Messaging, Abstinence Promotion, Documentation, Advice Provision, Diet Recording, Disease Control Assessment, Checking, Symptom Exploration, One-on-One Coaching, Incentive Mechanisms, Assessing and Adjusting, Self-Care, Supervision, Self-Directed Learning, Symptom Recognition, Treatment Option Understanding, Rehabilitation Plan Adjustment, Notification System, Practical Tips, Simulated Exposure, Record Keeping, Reconciliation, Positive Refocusing, Goal Re-Engagement, Activity Integration, Content Framing, User Experience Optimization, Adherence Improvement, Record Sharing, Behavioral Recording, Cognitive Behavioral Approach, Replacement Strategies, Media Hashtag Classification, Acceptability Assessment, Interactive Access to Procedures, Positive Reinforcement, Usability Testing, Electronic Health Record Integration, Reality Acceptance, Preparation for Change, Co-Creation, Participatory Design, Optional Referral, Content and Functionality Incorporation, Data Quality Check, Gift Card Reward, Alerting, Payment Incentives, Tracking Calendar, Relapse Prevention, Outreach, Social Norm Establishment, Repetitive Viewing, Risk Assessment, Staged Training Program, Gaming Elements, Graduated Immediate Response, Structured Monitoring, Quizzes, Goal Reflection, Self-Reflection, Health Report Generation, Performance Ranking, Personal Medical Record, Concentration Challenges, Self-Advocacy, Lottery Incentives, Clinical Management and Coordination, Family Support, Facilitator Identification, Adaptive Messaging |
